# Evaluation of Healthcare-Associated Infections and Daily Bathing Compliance on BMT Unit after Introduction of 2% CHG Wipes

**DOI:** 10.1017/ash.2025.306

**Published:** 2025-09-24

**Authors:** Richard Hankins, Missy Kneifl, Miranda Neumann, Elizabeth Grashorn, Kelly Cawcutt

**Affiliations:** 1 University of Nebraska Medical Center; 2 Nebraska Medicine; 3 Nebraska Medicine; 4 Nebraska Medicine; 5 University of Nebraska Medical Center

## Abstract

**Background:** Healthcare-associated infections (HAI) and multi-drug resistant organisms (MDRO) are a significant cause of morbidity and mortality in the hospital setting. Bacteria often colonize a patient’s skin and can become a source of infection. Bathing patients with chlorhexidine (CHG) has been shown to decrease colonization with MDROs, central-line associated bloodstream infection (CLABSI), and HAIs. The best method for applying CHG remains unknown and hospitals continue to employ different methods of CHG bathing. **Methods:** This was a nursing led quality improvement project due to staff shortages to reduce the workload burden of a 36-bed bone marrow transplant (BMT) and medical oncology unit. Prior to October 2023, all patients on the unit received a daily CHG bath with 4% CHG solution, which was the standard of care in the rest of the hospital. Beginning in October 2023 patients who were admitted or transferred to the unit had an initial bath with a 2% CHG wipe. Patients would then receive a daily CHG bath with a 4% CHG solution. If a patient were to refuse a bath with the 4% CHG solution, they would be offered a bath with the 2% CHG wipes. The goal of the quality improvement project was to improve compliance with daily CHG bathing, and to reduce HAIs. A pre/post analysis was performed assessing daily bathing compliance and HAIs and MDROS on the BMT unit for the 9 months before and after the intervention. **Results:** From January 2023 through September 2023, there were 9187 patient days on the unit, with 26 documented mucosal barrier injury (MBI) CLABSI (2.83 per 1000 patient days), 2 MRSA bloodstream infections, and 3 VRE bloodstream infections. From October 2023 through June 2024, there were 9176 patient days on the unit with 19 documented MBI CLABSI (2.07 per 1000 patient days), no MRSA bloodstream infections, and no VRE bloodstream infections. Daily CHG bathing compliance increased from 75% in the 3 months prior to the intervention, to 82% after the intervention. **Conclusion:** Utilizing a mixed method daily CHG bathing regimen that includes 2% CHG wipes increases compliance of daily CHG bathing, and decreases HAIs and MDROs compared to a regimen with only 4% CHG solution. HAI reduction could be accomplished through reducing microbial colonization on the skin, or possibly simply by increasing overall compliance. Further study on this could evaluate the reduction in workload burden, cost-effectiveness, and reduction in HAIs.

